# The contingent impact of artificial intelligence on teaching effectiveness: a meta-analytic review of boundary conditions and moderating factors

**DOI:** 10.3389/fpsyg.2026.1744690

**Published:** 2026-04-29

**Authors:** Jie Cai, Jiaqi Zhu, Xinmeng Tang, Guanyi Li

**Affiliations:** School of International Economics and Business, Nanjing University of Finance and Economics, Nanjing, China

**Keywords:** artificial intelligence application, meta-analysis, reconstruction of teacher’s role, teaching reform, technology-enhanced learning models

## Abstract

The digital transformation of education is reshaping pedagogical landscapes and driving the shift toward smart teaching. This meta-analysis examines the impact of Artificial Intelligence (AI)-enabled teaching on intelligent teaching assessment, synthesizing evidence from 35 pre-test-post-test designs and 37 post-test studies. Results show that AI-driven interventions yield significant positive effects, with the post-test effect size (g_p = 0.586) exceeding the pre-test-post-test gain (g_delta = 0.136), primarily due to stricter baseline controls in the latter. Heterogeneity is mainly driven by AI type and results direction, while methodological factors have minor influence. Low publication bias and robust analyses support the reliability of findings, underscoring the importance of appropriate AI application. These insights provide empirical support for reconfiguring teachers’ roles and implementing data-driven instruction, offering strategic guidance for teaching process redesign and the enhancement of teachers’ digital competencies via personalized and adaptive learning.

## 1 Introduction

As a cross-disciplinary field integrating computer science, neuroscience and mathematics, Artificial Intelligence (AI) aims to develop systems that simulate and extend human intelligence. As a key technological force, AI is fundamentally changing the structural conditions of various fields in social production and life. In sectors such as industry, healthcare, transportation, and finance, AI has improved efficiency, service quality, and risk management through automation, diagnostics, routing, and predictive analytics (Bajwa et al., 2021; Fazlollahi et al., 2023; Jing et al., 2018). These applications share core characteristics, such as data-driven decision-making, personalized adaptation, and enhanced efficiency, suggesting that similar AI mechanisms can be transferred to address the unique challenges of education. In the educational ecosystem, AI shows a trend of greater potential for transformation compared to traditional teaching methods. Unlike traditional teaching methods, AI-assisted systems can establish an adaptive learning environment by leveraging interdisciplinary digital resources (i.e., libraries of materials integrating multiple disciplines) and multimodal content (including text, images, videos, and other forms). These systems use a data-driven personalized approach to plan customized learning paths for learners, overcoming the limitations of the “one-size-fits-all” educational model. This means that AI can improve teaching efficiency, promote the transformation of educational forms towards personalization, dynamism, and intelligence, and help establish sustainable personalized learning models to meet the diverse needs of future learners (Chen et al., 2020.).

The application of AI in educational models mainly presents three interrelated functional dimensions, namely instructional resource development, learning scenario construction, and personalized solution design (Ahmad et al., 2022; Bayly-Castaneda et al., 2024; Cao et al., 2025; Fan and Jiao, 2021). In instructional resource development, AI’s material collection and organization capabilities ease teachers’ selection workload, lower the barrier to technology integration, and enhance the alignment between resources and learning goals. In the learning scenario construction, the learning environment assisted by AI, with its flexible access mechanism, breaks through the limitations of time and space, and combined with teaching methods such as gamification and flipped classroom, effectively enhances the participation degree and exploration behavior of learners. In the personalized solution design, relying on the personalized learning driven by AI, using adaptive algorithms, teaching plans are generated based on the learner’s profile, and can be adjusted in real time according to the individual’s progress trajectory, thereby achieving precise adaptation (Walkington and Bernacki, 2020).

The effectiveness of these innovative AI teaching models is becoming increasingly remarkable when examined through the lens of the above three application dimensions in relation to how they tackle long-standing challenges in education. These challenges include growing student diversity, rising demand for personalized teaching, and the need for pedagogy to keep pace with rapid socio-technological change, which raise the bar for educational model adaptability. In this context, AI-driven models have brought critical opportunities for transforming teaching practices. Data-driven teaching methods can analyze the diversity of student characteristics and provide scientific support for educational decisions, which is conducive to responding to the variations presented by student group differences (Hase and Kuhl, 2024). The automation of administrative tasks enables teachers to reduce the time spent on routine work, thereby allowing them to devote more energy to interacting with students. Personalized learning support systems can assist in differentiated teaching based on learners’ abilities and progress differences, directly accommodating the specific needs of personalized teaching (Cao et al., 2025). These three types of applications enhance teacher operational efficiency and promote student engagement by improving evidence-based decision-making, creating more time for direct teacher-student interaction, and building student adaptability. In terms of personalized solution design, the design of personalized paths is particularly crucial. AI can generate teaching plans based on learner profiles and make dynamic adjustments as individuals progress, providing unique solutions for implementing differentiated teaching and providing precise feedback.

Although AI has the potential for transformation, its application in education still faces many challenges, demonstrating its “double-edged sword” nature (Alasadi and Baiz, 2023; Hwang et al., 2020; UNESCO, 2021; Aung et al., 2021; Zhu et al., 2025; Ye et al., 2025). Firstly, excessive reliance on AI may weaken students’ cognitive initiative and reduce teachers’ autonomy, as teaching decisions can be dominated by algorithms, limiting professional judgment (Steele, 2023). For example, generative AI’s writing assistance may reduce students’ learning efforts (Li and Ironsi, 2024). Secondly, the accelerated pace of technological iteration has raised the requirements for educators in digital skills, creating a gap between technical mastery and classroom implementation capabilities, which limits the effective implementation of AI. Thirdly, the risk of algorithmic bias is becoming increasingly evident: when AI systems are trained on historical data reflecting social inequality, they may acquire and amplify such biases (HolonIQ, 2023). For instance, in teaching basic concepts such as computational thinking, poorly designed usage goals and methods based on students’ cognitive stages can make generative AI tools less effective than traditional methods (Hsu and Hsu, 2025). These challenges are interrelated: insufficient digital skills may lead teachers to use AI less appropriately, thereby increasing the risk of algorithmic bias or excessive reliance. This demonstrates that the effectiveness of AI depends on appropriate application. Misuse may not only fail to achieve the intended goals but also cause negative effects.

The aforementioned dual nature, such as varied AI efficacy and stage-dependent contextual effects, renders research results susceptible to sample characteristics and design, leading to inconsistent findings. To integrate these pieces of evidence and address the heterogeneity issue, this study adopts meta-analysis as the core method. The literature search is based on the Web of Science Core Collection, with a time span from 2015 to 2024, and the keywords include AI, teaching, and experimental design. To ensure rigor, the quality of included studies was independently assessed using the Joanna Briggs Institute (JBI) quasi-experimental research quality checklist. This study uses the standardized mean difference (SMD) as the effect measure, establishes the “pre-test/post-test model” to evaluate the effect before and after the intervention, and the “post-test-only model” to assess the absolute effect after the intervention, thereby complementing each other to capture the short-term and long-term impacts of AI on teaching effectiveness. Through funnel plots and Egger tests, the symmetry of the research distribution and potential publication bias are examined, and the heterogeneity of the studies is quantified using the I^2^ index. Moderator analysis was used to identify key heterogeneity sources (e.g., AI tool types, educational stages) and clarify boundary conditions and optimization paths for AI application in education.

Based on the above analysis, this study has expanded in three aspects on the existing literature on the systematic evaluation of the impact of AI on teaching practice. Firstly, it corrects the evaluation bias of “overemphasizing students and neglecting teachers,” and establishes the core position of teachers’ teaching performance. Most previous studies have mainly focused on the direct effect of AI on students’ academic performance, but have neglected the value of teachers’ specific achievements (such as adjustment of teaching strategies, role transformation, etc.). This study is the first to regard teachers’ teaching performance as the core evaluation indicator, and conducts a comprehensive analysis of 21 quasi-experimental studies, thereby confirming that AI has an overall positive impact on teaching performance. The value of AI is not only to help improve students’ learning outcomes, but more importantly, it can promote the teacher’s role from a simple “knowledge transmitter” to a “learning designer,” re-shaping the dynamic characteristics of the teaching process and transforming technical tools into strategies that can be integrated into daily teaching.

Secondly, it fills the gap in the perspective of “technology/student characteristics determining heterogeneity,” and reveals the mediating regulatory role of teachers. Most previous studies have simply attributed the heterogeneity of AI effects to technical characteristics or student characteristics, but have ignored the mediating regulatory value of teachers as the bridge between technology and learning. This study regards teachers’ abilities (including technical literacy, teaching design ability) as key moderating variables, under the guidance of situational learning theory (emphasizing “realistic context creation”) and Csikszentmihalyi’s flow theory (emphasizing “optimal experience conditions”). This translates abstract theories into measurable moderators: capable teachers operationalize them by creating authentic settings for situated learning and fostering conditions that support learners’ flow. Through heterogeneity analysis and moderating variable testing, this study validates quantitatively: the moderating effect of teachers’ technical literacy is significant, and low teachers’ technical literacy will expand the variance of teaching performance and directly increase the difference in teaching performance. Advances in technology do not by themselves guarantee gains in teaching effectiveness; the realization of AI’s potential hinges on teachers who are adequately prepared.

Finally, this study has made a significant breakthrough, breaking away from the limitations of “single technology, single stage” research. It provides empirical evidence regarding the applicability of different AI tools in various educational stages, namely primary school, secondary school, vocational education, and higher education. While previous studies focused only on the isolated effects of specific technologies or stages, hindering the identification of cross-context adaptation patterns, this study conducted a systematic analysis of the matching relationships between specific AI types and teaching objectives, contexts, and learner characteristics in each educational stage. It was found that the application effects of AI are regulated by learners’ cognitive development and subject demands, with the collaborative effect between AI application and the dominant cognitive patterns in each stage serving as the primary driver of effect differentiation, which clarifies the boundary conditions of AI use in different educational scenarios and provides a reference for optimizing the selection and implementation of AI tools across stages.

Considering that AI in education holds transformative potential but also faces numerous challenges, and that existing research has significant gaps in teacher teaching performance, technology-effect relationships, and cross-stage adaptability, this study intends to integrate existing evidence through meta-analysis to answer the following three research questions: First, can the integration of AI technology effectively improve teachers’ teaching performance? Second, is there a necessary connection between technological progress and the improvement of teaching effects? Third, how do the interactions between different educational stages, namely primary school, secondary school, and higher education, and different types of AI tools affect teachers’ teaching performance?

## 2 Methodology

Meta-analysis, first proposed by Glass in 1976, is a statistical method that employs quantitative approaches to synthesize the results of multiple existing studies in a specific field, featuring systematicity and strict literature screening criteria. This study conducted the meta-analysis in accordance with the Preferred Reporting Items for Systematic Reviews and Meta-Analyses (PRISMA) framework (Moher et al., 2009; Page et al., 2021) to ensure the transparency of the research process, the reproducibility of results, and the completeness and standardization of reporting.

### 2.1 Data sources and search strategy

This study aims to comprehensively explore the impact of artificial intelligence (AI) on teachers’ teaching effectiveness through meta-analysis methods. The first step is that we mainly search for relevant literature on the impact of AI on teachers’ teaching effectiveness in recent years from the Web of Science (WOS), Scopus, and ERIC databases. The search time span is set from 2015 to 2024. Corresponding search terms are employed in accordance with the specific syntax requirements of each database. To comprehensively cover the main application technologies of artificial intelligence in the field of education, the search terms adopted cover multiple research dimensions related to AI technology and education and teaching. The specific search terms include “Artificial Intelligence” or “AI” or “Machine Learning” or “Deep Learning” or “Neural Networks” or “Natural Language” “Processing” And combine “teacher” or “instruction” or “teaching practice” or “pedagogical practice” or “classroom practice” or “pedagogy” strategies or instructional design. In addition, to ensure comprehensive coverage of the perspective of teachers’ teaching research, we have included terms related to experimental design: including “empirical,” “experimental,” “controlled,” or “interventionist,” and are accompanied by “control group,” “experimental group,” “intervention group,” “comparison group,” or “placebo group.” Furthermore, as this study integrates multiple independent studies through meta-analysis, it is necessary to conduct a comparative analysis of the learning outcomes of the experimental group and the control group before and after the test. Therefore, the search formula is further limited to ensure the accuracy of the results: excluding “review,” “conference,” “theoretical,” “meta-analysis,” “systematic review,” or “scope review.”

### 2.2 Literature screening criteria

A preliminary search in this study obtained 1,681 English literatures. To further conduct refined and in-depth research and screen literature samples that meet research requirements, the following literature screening criteria are formulated:

Provide sufficient information and statistical data for the meta-analysis;

Focus on the effects of key variables on teaching effectiveness;

Include comparative analyses of pre-test and post-test learning outcomes between experimental and control groups;

Be assessed as high-quality studies using the University of the West of England Critical Appraisal Framework for Research Papers (Moule et al., 2003). This framework involves two reviewers conducting a comprehensive evaluation of six components: introduction, research methods, data collection and analysis, ethics, results, and conclusions. Both reviewers have extensive experience in literature quality assessment, scored each study independently, and submitted the results to the researchers. In case of disagreement between the two reviewers, a third reviewer was consulted to make the final decision.

Studies were excluded if they met any of the following criteria:

Not written in English;

Unable to provide sufficient data for the meta-analysis;

Without a controlled experimental design;

Assessed as low-quality studies by two experienced reviewers using the aforementioned University of the West of England framework;

Are review articles, theoretical studies, conference abstracts, or meta-analyses.

Any disagreements between the two reviewers during literature screening or quality appraisal were resolved by consulting a third experienced reviewer for a final decision.

### 2.3 Data screening

**FIGURE 1:**
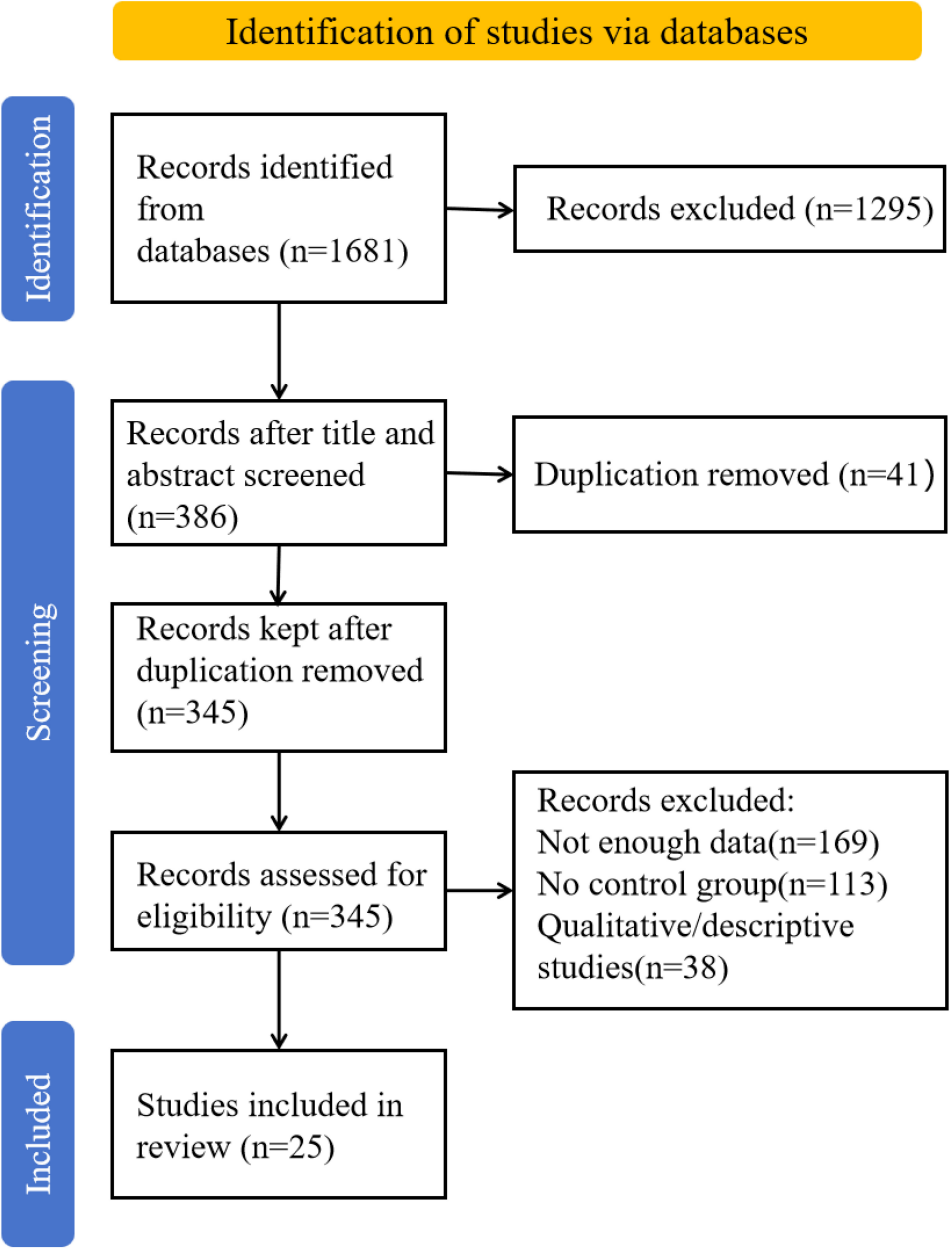
Data selection process.

As shown in Figure 1, data screening mainly consists of three steps: identification, screening, and inclusion. Specifically, it refers to the process of screening the data source after determining it, eliminating duplicate and irrelevant literature, and ultimately including it in the sample. In the identification stage, 1,681 records were extracted from the database based on the aforementioned retrieval keywords. During the screening stage, an independent working mode was adopted. Researchers conducted blind screening of the database based on the inclusion criteria. After screening the article titles, abstracts and sources, 386 relevant documents were finally determined. After removing duplicates, 345 studies were retained. Subsequently, 169 studies with missing data, 113 studies without control groups, and 38 other qualitative (descriptive) studies were excluded, ultimately resulting in 25 studies being included for subsequent meta-analysis. Table 1 presents a detailed analytical summary of the 25 articles included in this study.

**TABLE 1:** Articles included.

Authors	Country	*N*	stage	Intervention type	Intervention duration	Dependent variable	AI type	Test used	Results
Wang et al.	Taiwan, China	72 CG = 36 EG = 36	P	Ap	5 weeks	MQ, PE	Ap	CT, I	↑
Medina Herrera et al.	Mexico	255 CG = 93 EG = 162	U	VR	10 weeks	SA	VR/AR	CT	↑
Da Teng et al.	China	60 CG = 30 EG = 30	U	Ap	6 weeks	SA, MQ,PE,Sa	Ap	CT, PQ, MQ, Sa	↑
Yongliang Wang et al.	China	113 CG = 56 EG = 57	U	Ap	4 weeks	PE, MQ, Sa	Ap	PQ, MQ, Sa	↑
Sanal Kumar et al.	India	195	U	Ap	6 months	SA	Ap	CT, MQ, Sa	↑
Wu et al.	China	31 CG = 16 EG = 15	U	Ap	16 weeks	SA	Ap	PQ,I,CT	↑
Li et al.	China	58 CG = 29 EG = 29	U	Ap	14 weeks	SA	Ap	CT,I	NS
Xu et al.	China	54 CG = 27 EG = 27	TVS	VR	8 weeks	SA, LE, LI	VR/AR	CT, PQ	↑
Rodríguez-Abad et al.	Spain	137 CG = 65 EG = 72	U	AR	3 h interactive session	SA, MQ, Sa	AR	CT, PQ, MQ	↑
Tang et al.	Hong Kong, China	72 CG = 28 EG = 44	U	MR	10 min	SA,SA,SA	MR	CT,CT,CT	↑
Chen et al.	China	47 CG = 21 EG = 26	P	AR	4 weeks	SA	AR	CT	↑
Melese Astatke et al.	Taiwan, China	40 CG = 20 EG = 20	S	VR	90 min	SA, MQ, PQ	VR	CT, MQ, PQ	↑
Huang et al.	Taiwan, China	43 CG = 22 EG = 21	P	AR	160 min	SA	AR	CT	↑
Hsu et al.	Taiwan, China	56 CG = 28 EG = 28	P	Ap	60 min 160 min 200 min	SA	Ap	CT,PQ	↑
Chang et al.	Taiwan, China	40 CG = 19 EG = 21	S	AR	18 weeks	SA	AR	CT	↑
Tarng et al.	Taiwan, China	60 CG = 30 EG = 30	S	VR	1 teaching experiment	SA, MQ, PQ, Sa	VR	CT, MQ, PQ, Sa	↑
Lin et al.	Taiwan, China	52 CG = 25 EG = 27	S	AR	45 min	SA,MQ,PE	AR	CT,PQ	↑
Marrahi-Gomez et al.	Spain	130 CG = 66 EG = 64	S	AR	4 weeks	SA,MQ	AR	CT,PQ,I	↑
Zhang et al.	China	70 CG = 35 EG = 35	TVS	Ap	8 weeks	SA	Ap	CT,I	↑
Huang et al.	Taiwan, China	43 CG = 22 EG = 21	P	AR	160 min	SA	AR	CT,UO	↑
Ye et al.	China	111	S	Ap	11 weeks	SA,CT, MQ	Ap	CT,PQ	↑
Ulzhamal Konakbayeva et al.	Kazakhstan	37 CG = 11 EG = 13	U	Ap	8 weeks	SA,MQ	Ap	CT,PQ	↑
Stefano Scippo et al.	Italy	35 CG = 17 EG = 18	P	Ap	9 sessions (21 h)	SA,PE,Sa	Ap	CT,PQ	↑
Karakaya Özyer et al.	Turkey	94 CG = 66 EG = 28	U	Ap	2 h + online training	SA,MQ	Ap	PQ	↑
Meylani et al.	USA	591 CG = 295 EG = 296	S	Ap	2 seasons	SA	Ap	CT	↑

### 2.4 Meta-analysis

This study adopted the Joanna Briggs Institute (JBI) Checklist to conduct independent quality assessment of the included literature, which was completed independently by two researchers. The checklist consists of nine items, and each item is rated as Yes, No, Unclear, or Not applicable based on the reporting of the literature. If there were discrepancies between the two researchers’ evaluation results, consensus was reached through discussion or by referring to a third party for arbitration. According to the number of “Yes” items, the risk of bias of the literature was divided into three levels: low bias (8–9 Yes), moderate bias (5–7 Yes), and high bias (≤4 Yes). The results of the quality assessment showed that among the 25 studies, one study (4.0%) had low bias, 16 studies (64.0%) had moderate bias, and eight studies (32.0%) had high bias. Detailed evaluation results are provided in the Supplementary materials.

In the meta-analysis process, the standardized mean difference (SMD) is used as the effect size indicator, and a pre-test-post-test model and a post-test-only model are constructed, respectively. Heterogeneity and publication bias are analyzed through funnel plots and Egger’s test. Our sample of 25 studies, resulting from a rigorous selection process, has demonstrated high reliability and robustness. Therefore, the sample size is sufficient for a meta-analysis to meet the standards of academic publications (Ahn and Kang, 2018; Carlson et al., 2023).

## 3 Results

### 3.1 Experimental literature summary

With the rapid development of artificial intelligence (AI) technology, its application in the field of education is profoundly reshaping teaching practices, driving a reconfiguration of the teacher’s role and a reimagining of teaching processes (HolonIQ, 2023; Luo et al., 2025). Drawing on 25 empirical studies, this paper systematically examines the impact of AI technologies—including generative AI, large language models, intelligent teaching assistants, augmented/virtual/mixed reality, and gamified learning—on teaching reform (Phanwiriyarat et al., 2025).

#### 3.1.1 Generative AI and large language models: reshaping personalized teaching and teachers’ working models

Generative AI has provided new tools for the innovative design of teaching content in instructional resource development and personalized solution design (Chai et al., 2025; Ellikkal and Rajamohan, 2025; Teng et al., 2025). Studies have shown that combining the images generated by generative AI with gamified learning can effectively enhance the computational thinking ability and AI literacy of primary school students, especially in algorithm thinking and “creating AI.” However, the research also mentioned that relying excessively on generative AI tools might weaken students’ in-depth exploration of basic computational thinking concepts. Therefore, it is necessary to maintain a balance with traditional teaching methods and adopt a phased introduction strategy: students first establish a basic understanding through traditional methods, and then use AI to support creative expression and efficient execution. This indicates that redefining the role of teachers in the stage of teaching digital transformation is of considerable significance. Teachers need to transform from simple knowledge imparter to designers, facilitators, and coordinators of AI application. Intelligent teaching assistants based on large language models have great potential in intelligent teaching models such as flipped classrooms. Taking the computer vision course as an example, the LLM teaching assistant integrating retrieval-enhanced generation and multi-agent collaboration framework can provide 24/7 personalized question answering, automatic assessment, content suitability checks, etc., improving students’ academic performance, participation, and satisfaction, and also reducing the burden on teachers in preparing personalized materials and providing immediate feedback. This demonstrates an initial form of human-machine collaborative teaching, in which AI undertakes tasks such as answering questions, conducting evaluations, and monitoring, which are scalable, while teachers focus on more profound activities such as inspiring, guiding, and emotional interaction (Chejara et al., 2024; Ericsson and Harwell, 2019; Sanal Kumar and Thandeeswaran, 2024). The same situation also exists in the field of English listening and speaking teaching, where the flipped classroom simulation established with AI has been proven to be effective in promoting students’ autonomous learning ability, academic performance improvement, and has also alleviated the burden on teachers in preparing personalized materials and providing immediate feedback (Phanwiriyarat et al., 2025; Sancar-Tokmak and Dagli, 2025; Wu and Wang, 2021). However, these applications also face challenges, such as the possibility that model outputs may overly simplify complex concepts, thereby leading to an excessive reliance on technology and potentially hindering the development of students’ critical thinking and independent problem-solving abilities. Therefore, improving teachers’ digital literacy is extremely crucial. Educators need to master teaching strategy design to ensure that AI tools are used as auxiliary means to enhance rather than replace core cognitive processes. Overall, both types of AI applications require teachers to use digital literacy design strategies to achieve the deep integration of technology and teaching tools.

#### 3.1.2 Intelligent conversational agents and adaptive learning systems: promoting language skills development and targeted instructional interventions

AI-driven chatbots (Wang and Xue, 2024) have emerged as effective tools for personalized instruction in the field of language education (Olugbade et al., 2024). Research on Chinese EFL learners indicates that a 4-week supplementary teaching programme utilizing AI chatbots such as TalkAI and SpeakG can significantly enhance students’ academic engagement across behavioral, cognitive and affective dimensions (Liu et al., 2023). The mechanism behind this lies in the provision of an environment offering immediate, personalized feedback and interaction. In writing instruction, AI systems that combine automated assessment with peer assessment are more effective than automated assessment alone in improving students’ writing performance, fostering critical thinking, and reducing writing anxiety (Hernon et al., 2023). This suggests that the integration of intelligent assessment systems with social learning mechanisms can create a richer learning experience. However, some studies have also pointed out that, in the development of writing skills among pre-service science teachers, the use of ChatGPT alone did not yield more significant results than traditional methods combined with critical digital literacy; it may even foster a negative attitude of dependence on technology among students. This once again emphasizes that the integration of technology must be based on clear instructional process redesign and data-driven teaching, rather than blind application (Pijeira-Díaz et al., 2024; Zhang et al., 2024).

In the field of STEM education (Astatke et al., 2024, Wang et al., 2022), adaptive learning systems provide customized content by analyzing learner characteristics (Muskania et al., 2024). In programming education, an adaptive video learning system based on a decision tree model (VPTH) can match different video design patterns to students’ learning style preferences, thereby significantly improving their academic performance. A learning approach combining mind maps with GenAI chatbots has also been shown to effectively improve secondary school students’ programming performance, computational thinking, and self-efficacy in programming. These examples demonstrate how adaptive learning systems can achieve precise teaching interventions through education data-driven analysis, thereby supporting teachers in delivering differentiated instruction.

#### 3.1.3 Extended reality technologies and gamified learning: creating immersive contexts and optimizing the learning experience

Augmented reality (Xu et al., 2023), virtual reality (Tarng and Pei, 2023) and mixed reality (Tang et al., 2020) technologies provide powerful support for learning scenario construction by creating immersive learning environments in the re-engineering of teaching processes (Chan et al., 2021, Chen et al., 2024; Kuhn et al., 2016; Makransky and Petersen, 2021; Chang et al., 2020; Lin et al., 2020). In primary school science inquiry-based learning, teaching methods that combine AR with concept maps not only significantly improve academic performance but also optimize students’ learning behavior patterns, reduce ineffective reading time and enhance problem-solving efficiency. In health education, the introduction of AR-based board games has significantly improved students’ learning outcomes, positive emotions and sense of immersion (Gündüz and Akkoyunlu, 2020; Pujiastuti and Haryadi, 2023; Huang et al., 2025; Marrahi-Gomez and Belda-Medina, 2024). In English grammar learning, AR-enhanced classroom settings can effectively boost students’ motivation (ARCS model) and learning outcomes. In quantum mechanics, dance training, leg ulcer care and landscape architecture education, XR technologies have been shown to significantly improve learning outcomes and experiences by enhancing spatial understanding, providing real-time feedback and reducing cognitive load. A meta-analysis further confirmed that STEM education based on digital games has a moderate positive effect on student achievement. These technologies visualize abstract concepts and make complex processes interactive, enabling teachers to design intelligent teaching models that are difficult to achieve in traditional classrooms (Chiu, 2021; Korlat et al., 2024; Maldonado et al., 2024; Sriadhi et al., 2022; Gregorčič and Torkar, 2022; Rodríguez-Abad et al., 2022; Medina Herrera et al., 2024).

#### 3.1.4 Comprehensive discussion: challenges, integration pathways and the core role of teachers

In summary, these studies collectively paint a multifaceted picture of AI-enabled educational reform (Ren et al., 2025; Twabu, 2025). Various AI technologies support educational reform through different pathways: generative AI and LLMs focus on instructional resource development and personalized interaction; intelligent conversational agents and adaptive systems focus on skills training and precise feedback; XR and gamification focus on learning scenario construction and experience optimisation. Their shared objective is to achieve personalized solution design and the digital transformation of education.

However, the research also consistently highlights the challenges faced: The risk of student over-reliance: this may undermine deep thinking, exploratory learning and critical thinking. Balancing teaching ethics and effectiveness: This involves academic integrity, content bias, and the potential negative impact of technology use on the mastery of fundamental concepts. Teacher professional development needs: Teachers require training in the effective integration of AI to avoid sliding from complete resistance to over-reliance. Technical limitations: Such as the accuracy of output content and the oversimplification of complex problems (Ng et al., 2024; UNESCO, 2021).

Consequently, successful AI-driven educational reform is not merely a matter of superimposing technology, but requires the re-engineering of teaching processes and the reconfiguration of the teacher’s role. Ultimately, a balanced educational pathway should be established, combining automated tools with project-based activities that require in-depth exploration and knowledge construction, in order to maximize student learning outcomes (Fullan, 2020). In this context, teachers’ instructional design capabilities and professional judgment remain central.

### 3.2 Qualitative meta-analysis

**FIGURE 2:**
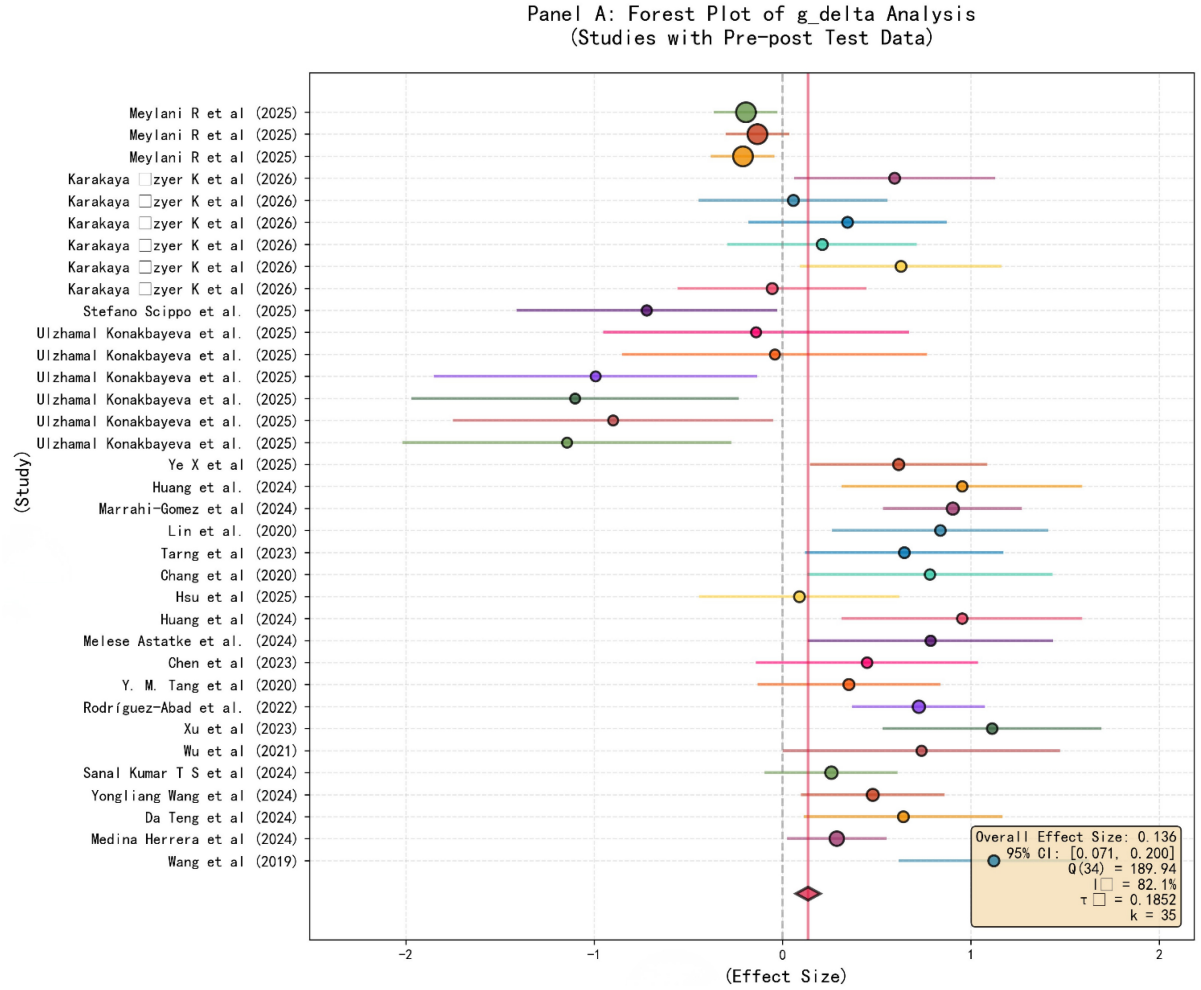
Pre-post difference effect size(g_delta) forest plot.

**FIGURE 3:**
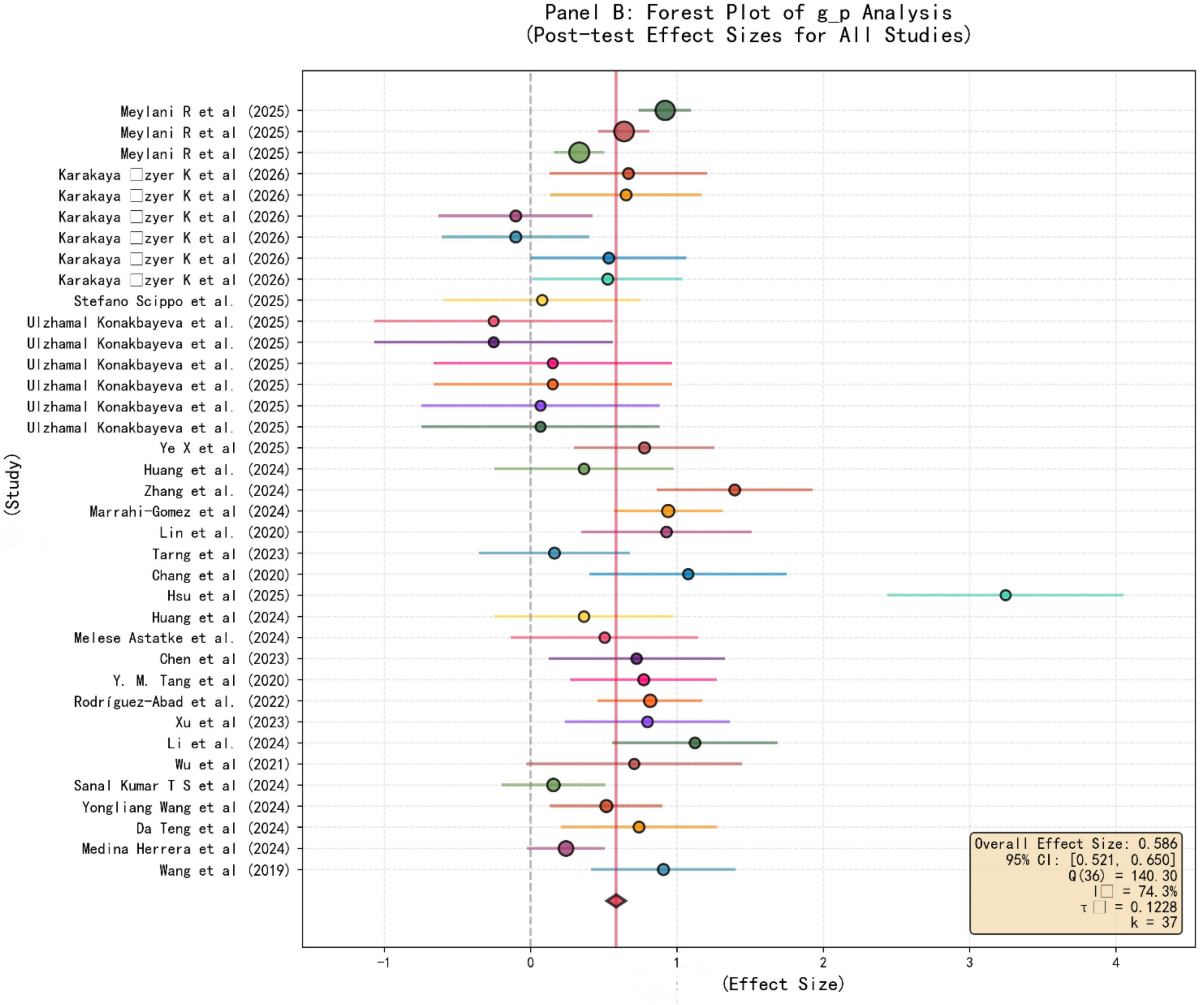
Post-test effect size(g_p) forest plot.

In the forest plot of the results of this meta-analysis, the forest plot of Figures 2, 3 present the evidence of the impact of AI-assisted teaching on teaching reform, depicting the standardized mean differences under different teaching scenarios and research designs. Figure 2 only included k equal to 35 studies that adopted a strict pretest-posttest design, and the cumulative analysis yielded a total effect size of 0.136 (95% confidence interval [0.071, 0.200], *p* < 0.001), which had a certain effect but was relatively small (i.e., the average increase was limited). The diamond mark at the bottom of the forest plot presented a positive point estimate, indicating that from intelligent tutoring systems to data-driven teaching and other AI intervention measures, compared to traditional methods, it can improve teaching effectiveness. The high heterogeneity situation was (Q = 189.94, df = 34, I^2^ = 82.1%, τ^2^ = 0.1852), which indicated that the effectiveness of teaching digitization varied greatly in different environments, and this could reflect the complex interaction relationship between technical tools and classroom dynamics. This means that the actual exertion of technical advantages is limited by classroom conditions, and the effect size distribution was relatively wide (−2 to 2), which further confirmed that the intelligent teaching model has certain potential to improve the effect compared to traditional teaching, but the actual effect is determined by specific contextual variables. Because the actual exertion of technical advantages is constrained by classroom dynamics and implementation conditions, this difference indicates that redesigning the teaching process is necessary to bridge the gap between technical advantages and classroom reality, that is, merely introducing AI cannot universally improve teaching quality, and it also requires a transformation of teaching strategies. Expanding the analysis scope to include all posttest data from the studies, Figure 3 presented a more robust comprehensive analysis result for k equal to 37 studies. The overall effect size g_p was 0.586 (95% confidence interval [0.521, 0.650], *p* < 0.001), showing an upward trend. The effect size distribution shifted to the right, with some study values exceeding 2, indicating that when evaluating the final outcome of AI-assisted teaching, the scale of the intervention effect was larger compared to the longitudinal benefits in Figure 2. Although the heterogeneity slightly decreased (I^2^ = 74.3%, τ^2^ = 0.1228), the significant difference still existed, indicating that the success of personalized and adaptive learning environments and the reshaping of the teacher’s role, as well as the improvement of digital capabilities, are closely related. The comparison between the two panels highlighted that although AI can establish infrastructure for intelligent teaching assessment and human-computer collaborative teaching, the benefits it brings to achieve this need to rely largely on the extent to which the implementation plan is faithfully executed and the teacher’s ability to navigate in complex data environments. It is important to note that the significant effect has obvious heterogeneity behind it, indicating that the effect is regulated by multiple factors, so further exploration of subject-specific adaptability, modern classroom social-technical dynamics, and other regulatory factors is necessary. The significant effect size of Figure 3 further confirmed that AI is a key factor driving teaching reform, but the inherent heterogeneity also raises a warning that further exploration of the above regulatory factors is necessary. Relying solely on the introduction of technology is not enough to ensure continuous improvement of educational quality; more attention should be placed on the cultivation of teachers’ digital capabilities, so that teachers can maintain sustainable and effective human-computer collaboration in the future intelligent teaching model.

### 3.3 Moderator analysis

**FIGURE 4:**
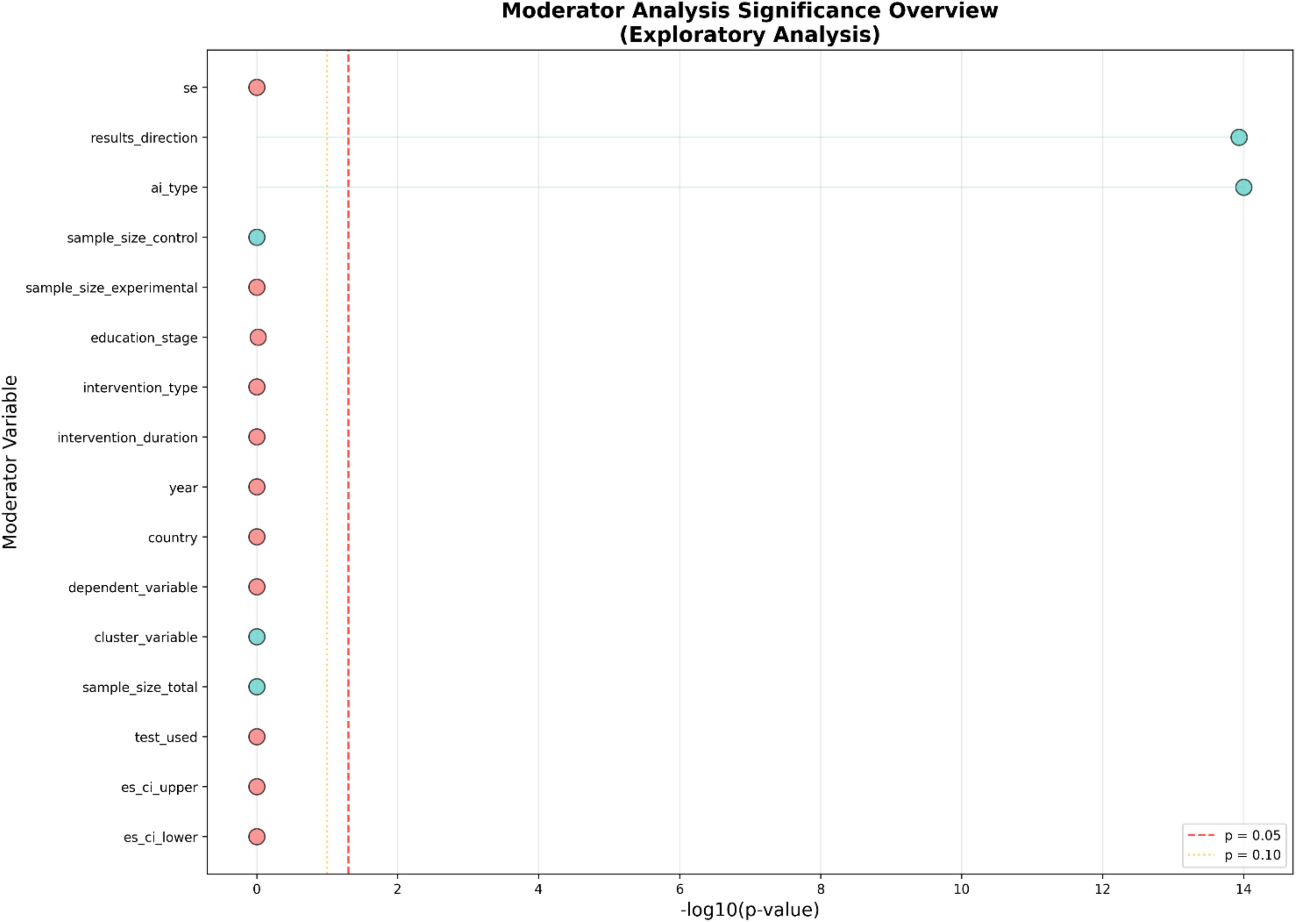
Moderator analysis significance overview (exploratory analysis).

In the context of the digital transformation of teaching, there are inherent differences in the research results, which require rigorous exploratory analysis to clarify the boundary conditions and moderating factors that affect the effectiveness of AI-assisted teaching interventions. By converting the distribution of −log_10_(*p*-values) into a range of potential moderating factors, the importance of the moderating factors is summarized into a statistical framework to distinguish which variables fundamentally alter the impact of intelligent teaching models on teaching outcomes. In Figure 4, the vertical axis presents a complete list of various moderating factors ranging from research design parameters to sample characteristics, while the horizontal axis quantifies the statistical significance. The red and yellow dashed lines, respectively determine the critical thresholds of *p* = 0.05 and *p* = 0.10. The empirical results show that the moderating effects of the studied variables are significantly different. The most prominent ones are the variables “results direction” and “ai type,” which become very powerful factors influencing the differences in effect size. The individual −log_10_(*p*-values) coordinates of these variables exceed 14, indicating a high statistical significance. This high statistical significance suggests that the specific configuration of teaching intervention based on AI, whether focusing on personalized and adaptive learning, intelligent teaching assessment, or teaching process reengineering, as well as the inherent directionality of the reported results, are the main determinants of the observed heterogeneity. These findings indicate that the success of the digital transformation of teaching is not a single phenomenon but largely depends on the technical architecture of the adopted AI system and the specific teaching goals it intends to promote. This also highlights that to understand the mechanism of different AI types, it is necessary to more carefully examine how they drive the reconfiguration of the teacher’s role.

Apart from the main driving factors, this analysis further illustrates the subtle role of methodological limitations and environmental factors in shaping the pattern of the effectiveness of AI-assisted teaching. Although most variables such as “experimental sample size” (sample_size_experimental), “education stage” (education_stage), and “intervention type” (intervention_type) did not reach the threshold of *p* = 0.05, this indicates a relative lack of moderating effects in the current model. However, the variable “sample_size_control” shows a statistically significant moderating effect, with its −log_10_(*p*-value) approaching the level of 1.0. This means that the size of the control group in the experimental design will affect the precision and magnitude of the observed effect size, highlighting the importance of strict experimental control when verifying the impact of data-driven teaching. In contrast, “education stage” and “intervention type” do not have significance, indicating that the potential of human-computer collaborative teaching may be able to exceed specific educational levels or broad intervention categories, meaning that the optimization of the AI-based teaching process is universally applicable as long as the core technical factors are optimized. Because these variables have a relatively weak impact on the effect size, even in the non-significant situation, it can be inferred that their application scope is relatively wide. The convergence of these findings highlights the heterogeneity observed in the research, mainly due to the qualitative characteristics of AI application, the consistency of result reporting, and not the age or structural characteristics of the student population. For further improvement of intelligent teaching models and enhancing teachers’ digital capabilities, subsequent research is necessary to prioritize subgroup analysis and meta-regression models focusing on “ai_type” and “results_direction.” This effort can provide the necessary empirical details to explain the specific direction and intensity of these regulatory effects, thereby laying a more solid theoretical foundation for integrating intelligent teaching assessment and adaptive learning systems into modern classrooms.

**FIGURE 5:**
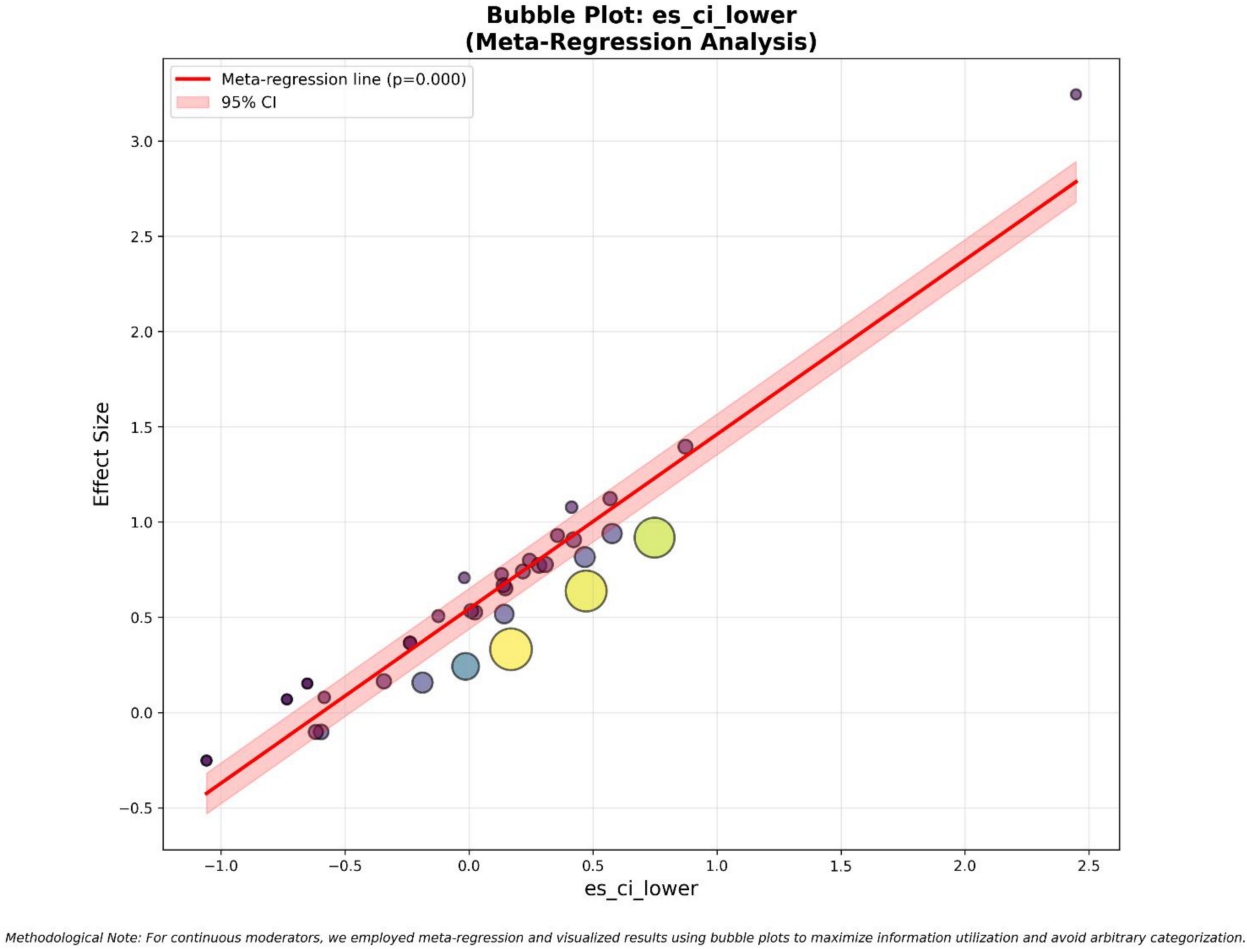
Bubble plot: es_ci_lower (meta-regression analysis).

Within the broader framework of the digital teaching transformation, empirical research on the effects of AI-assisted teaching must employ complex statistical methods and cannot merely be limited to simple classification subgroup analysis. Instead, a continuous meta-regression framework should be adopted to maintain the fine integrity of the data. As shown in the first meta-regression bubble chart (where the lower limit of the effect size confidence interval [es_ci_lower] is regarded as the main moderating factor), the precision of the lower limit estimates in the study is positively correlated with the overall observed effect size of the intelligent teaching model. In Figure 5, each bubble represents an independent study, and its diameter is proportional to the reciprocal of the variance - thereby reflecting the relative weight and accuracy of the study - showing that the distribution range of the effect size is approximately −0.5 to 3.2, and the es_ci_lower value covers the continuous interval from −1.1 to 2.5. This red regression line has a positive slope, and its *p*-value is 0, indicating a robust linear relationship. Lower limit thresholds are generally associated with larger intervention effect scales, meaning that studies reporting conservative but still positive baseline effects often yield higher overall average effect sizes. When considering the reconfiguration of the role of teachers, this phenomenon is particularly prominent. As teachers transform from traditional lecturers to guides for personalized and adaptive learning, the stability of the lower limit estimates can be regarded as an indicator of the reliability of AI-driven intervention measures. There is an outlier with an effect size of approximately 3.2 and an es_ci_lower of 2.5, further indicating that a thorough redesign of the teaching process may produce transformative results beyond the conventional distribution. Using this meta-regression technique, this analysis avoids the problem of information loss in manual classification and provides a more detailed perspective on how the statistical boundaries of each study reflect the actual effectiveness of data-based teaching methods and the improvement of digital capabilities of teachers in different teaching environments.

**FIGURE 6:**
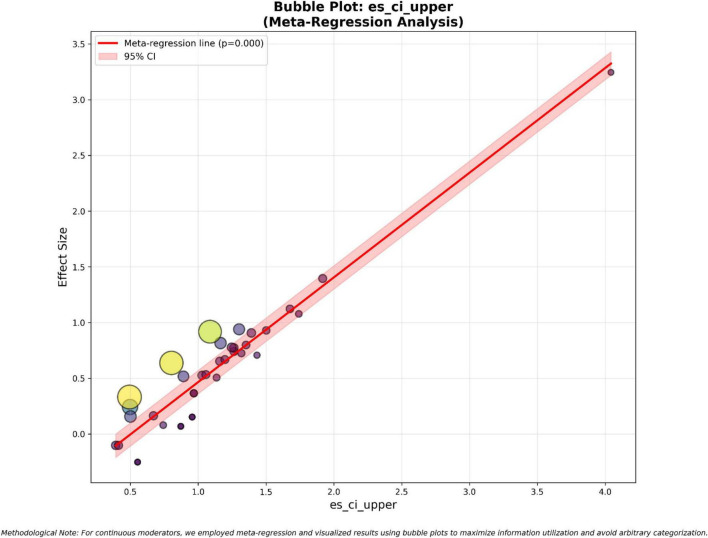
Bubble plot: es_ci_upper (meta-regression analysis).

At the same time, the second meta-regression graph focuses on the upper limit of the effect size confidence interval, which once again confirms the previously observed situation. This meta-regression graph shows a statistically significant and persuasive positive trend, with a *p*-value of 0.000. This trend verifies the overall impact of human and AI collaborative teaching. In Figure 6, the es_ci_upper value range is from 0.3 to 4.2, and the corresponding effect size is within the range of −0.3 to 3.3, indicating that as the potential upper limit of the intervention effect increases, the actual effect size will develop along the corresponding upward path. The red shaded area represents the 95% confidence interval of the regression line, its narrowness reflecting a high degree of fit and prediction reliability, indicating that the integration of intelligent teaching assessment and adaptive learning systems has brought continuous upward pressure on teaching quality. This relationship is not a simple mathematical result of the interrelation of effect size and its confidence interval, but an indication of a systematic transformation, meaning that the “optimal situation” of implementing AI is gradually becoming a common pattern in practice. The effect size is closely related to the precision and potential of the intervention, and the outlier shows that teacher capabilities and process reengineering can amplify the effect, from which it can be inferred that the collaboration of human expertise and AI constitutes the core driving factor of an efficient educational environment. The outliers observed in this graph have an effect size of 3.3, with the upper limit of the confidence interval being 4.2. This is a key data point for understanding the maximum potential of teaching digitization, indicating that when teachers’ digital capabilities are fully realized and the teaching process is redesigned comprehensively, there is a statistically significant change in teaching efficiency. This has a transformative impact on education. Ultimately, a comprehensive analysis of these two bubble charts confirms that the positive impact of AI on teaching reform is not a localized or sporadic phenomenon but a statistically reliable trend that emerges. The scale of this trend is proportional to the precision and potential of the intervention measures. These findings provide a rigorous empirical basis for the continuous digital transformation of teaching, highlighting the collaborative effect between human expertise and AI as the core driving factor of the next generation of personalized, data-driven, and efficient educational environments.

The dataset used in this study cannot currently support meaningful subgroup analyses of moderating factors. The main limitation is that only 37 studies were included, resulting in a relatively small sample size. Moreover, most of the categorized moderating variables lead to excessive subgroup divisions, with many subgroups containing only 1–2 independent studies. This directly causes severe statistical power deficiency, making it impossible to reliably identify or verify moderating effects. Even if subgroup analyses are conducted despite these limitations, the results would be unstable and lack statistical validity, making it difficult to draw reliable conclusions about the sources of heterogeneity.

### 3.4 Funnel plots analysis

**FIGURE 7:**
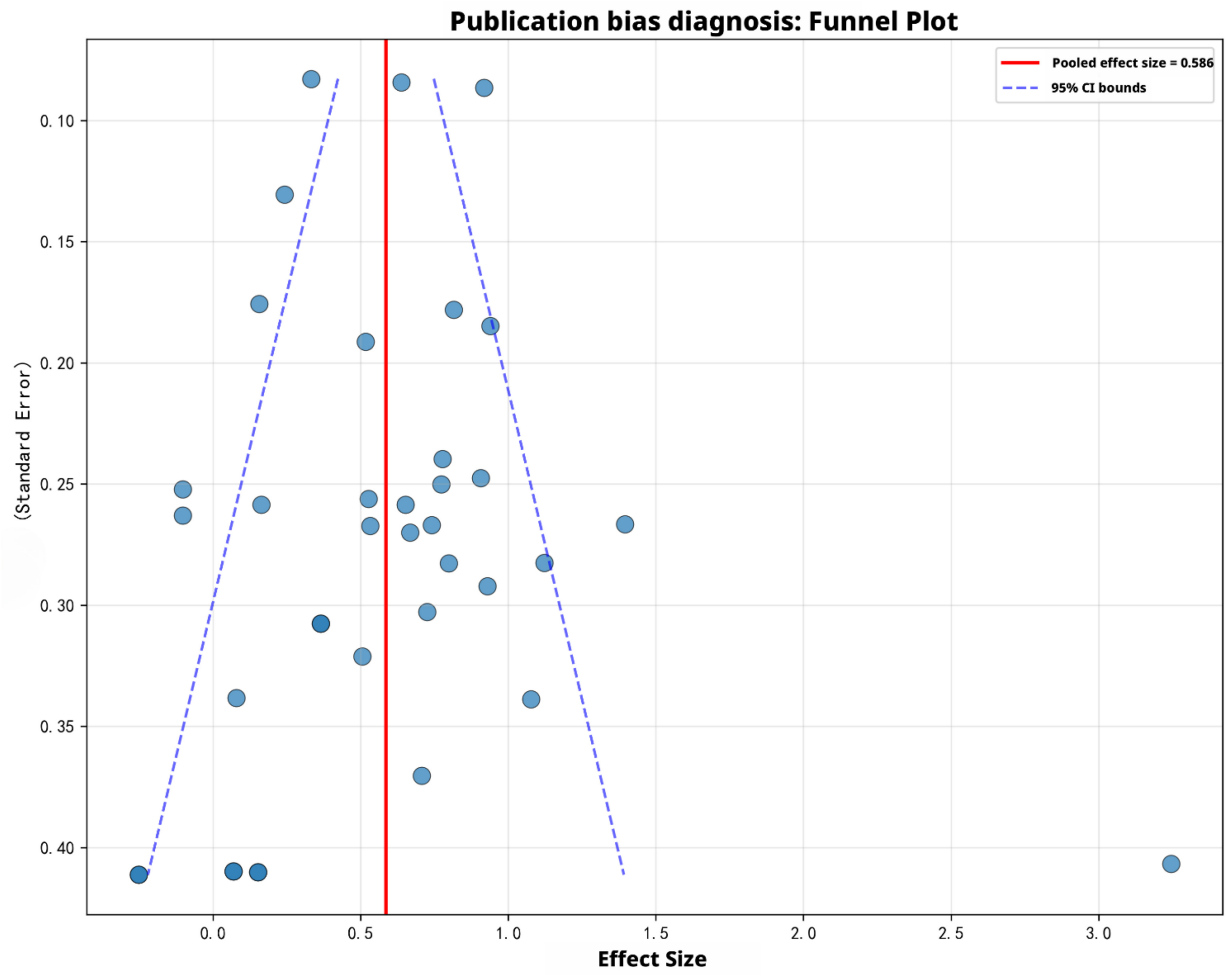
Funnel plot for publication bias assessment in meta-analysis.

When conducting a rigorous assessment of the digital transformation of teaching, the sieve diagram remains a key method and tool for identifying potential systematic biases in the effectiveness of AI-assisted teaching interventions. This visual approach can prevent the “file drawer problem,” which is the selective publication of results that are statistically significant or have significant numerical values, from adversely affecting the comprehensive analysis of evidence for intelligent teaching models. In Figure 7, the structure of the sieve diagram includes a horizontal axis representing the magnitude of the observed effect, ranging approximately from −0.5 to 3.3, covering a wide continuous range, and a vertical axis representing the standard error, serving as an inverse indicator of the precision and sample size of each individual study. The core feature of this framework is the red vertical line, which indicates a combined effect size of 0.586, meaning that integrating AI into teaching practice will have a moderate to strong positive impact. From a theoretical perspective, in the absence of publication bias, 37 independent studies (marked with blue circles) should present a symmetrical distribution around this mean. Among them, studies with higher precision, that is, smaller standard errors, are clustered at the top, while studies with lower precision, that is, larger standard errors, are more dispersed at the bottom, and all these studies are within the 95% confidence interval defined by the blue dotted line. However, a detailed analysis of the actual distribution reveals a significant deviation from this ideal symmetry, especially in the case of studies with low precision. Although studies with higher precision (SE < 0.2) are closely related to the 0.586 aggregated effect, studies with larger standard errors show a clear rightward bias. The lower right corner area shows a concentration of data points, including a significant outlier (effect size approximately 3.3). Given that larger standard errors typically indicate a smaller sample size, this phenomenon suggests that in the published literature, the proportion of highly effective small-scale studies is higher. In contrast, in the lower left corner area, a relatively obvious “gap” or “absence” can be observed, which should normally occur for those studies reporting no effect, a small effect, or negative effects. Such a distribution feature indicates that some research results have not been adequately reported. This visual asymmetry provides strong evidence that there is likely a publication bias phenomenon, meaning that current academic discussions may be systematically biased towards reporting the successful implementation of personalized and adaptive learning, but do not adequately report the challenges faced by teachers in redefining their roles or the lack of measurable improvements in student outcomes. This right-skewed distribution has a profound impact on the future development of data-based teaching methods, as it suggests that the overall combined effect size of 0.586 may overestimate the real impact of AI on teaching reform. This implies that the absence of studies with low precision and small effect sizes may lead to the underrepresentation of challenges faced by teachers in enhancing digital capabilities and the complexity of intelligent teaching assessment in cross-cultural contexts in the existing evidence. Such a distribution pattern is associated with the tendency of the academic community to prioritize the publication of studies with high impact or prominent results. An extreme outlier with an effect size of approximately 3.3 also requires a sensitivity analysis to determine whether it has excessively influenced the overall mean, or whether it represents a unique and outstanding example of AI teaching that is worthy of a qualitative study. Finally, although the existing evidence strongly supports the positive development trend of AI in the field of education, considering publication bias in an open and rigorous manner is crucial for providing a balanced empirical foundation for the continuous development of intelligent teaching models.

## 4 Discussion and conclusion

This meta-analysis combined 35 independent studies that employed pre-test and post-test designs and 37 studies with comprehensive post-test data to systematically evaluate the effects of AI-assisted teaching intervention measures on promoting the digital transformation of teaching. The research framework consists of four key points: the overall scale of teaching impact, the main drivers of observed research heterogeneity, the stability of the evidence base against publication bias, and the subsequent role of the reconstruction of the teacher’s role. The analysis summarized the results of forest plots, funnel plots, and meta-regression models. This discussion clarified the complex interplay between technological advantages and teaching implementation, laying an empirical foundation for the development of intelligent teaching models.

### 4.1 Does AI-enabled teaching significantly enhance instructional efficacy?

By analyzing the forest plot, it can be seen that the impact of AI on teaching outcomes shows an overall positive trend, although there are certain differences. In Figure 2, this graph focuses on presenting 35 intervention measures with strict pre-test and post-test data. The combined effect size calculated is g_delta = 0.136 [0.071, 0.200], *p* < 0.001, indicating a statistically significant but relatively mild positive effect. The high heterogeneity (I^2^ = 82.1%, τ^2^ = 0.1852) means that the effectiveness achieved by the teaching process reengineering largely depends on various background variables. In contrast, Figure 3 covers all 37 studies with post-test data, and its effect size g_p reaches 0.586 [0.521, 0.650], *p* < 0.001, reflecting moderate to strong teaching benefits. There is a difference between g_delta and g_p, highlighting the significance of baseline control. This is because the pre-test and post-test design controls the initial ability differences, so the net gain obtained after considering the starting level is more conservative. Although the intervention measures based on AI do improve performance, this comparison indicates that simply looking at the post-test results may overestimate the actual gains. These findings prove that when personalized and adaptive learning systems are integrated into the classroom, they not only supplement traditional methods but also fundamentally change the teaching trajectory. The shift from a “one-size-fits-all” data processing approach to a data-based teaching method enables real-time adjustments. However, the high I^2^ values in the two panels indicate that the educational benefits are not necessarily a result of the technology itself but are determined by the quality of human-machine collaborative teaching.

### 4.2 What are the primary drivers of heterogeneity in AI-driven instructional reform?

Through exploratory moderation analysis and meta-regression bar charts, it can be observed that some AI intervention measures have more obvious advantages compared to other measures, which is caused by specific factors. After analysis, it was clear that the main source of variation was ai_type and results_direction. The −log_10_(*p*) values of these two factors were far greater than the threshold of 1.3 (*p* < 0.05), indicating that they are the key determinants of the fluctuation in effect size. Specifically, the ai_type moderating factor indicates that different technical architectures such as generative AI and intelligent teaching assessment tools have different effects on the intelligent teaching model. The meta-regression analysis of the effect size confidence interval (es_ci_lower and es_ci_upper) presented a highly significant positive slope (*p* = 0), meaning that the precision of the research design, boundary conditions, and the reported success level are intrinsically linked. Sample size control and clustering variables showed marginal significance (*p* ≈ 0.10), indicating that methodological rigor and sampling strategies have an effect on the observed results. Variables such as publication year, geographical location, intervention duration, etc., had no significant impact on the effect. This suggests that time or geographical factors have a relatively small impact on the effect. The success of teaching digital transformation can be achieved in different times and regions, indicating that it is a global phenomenon. Its success is more dependent on the specific configuration of AI tools, the reengineering of teaching processes, and the coordination of teaching goals rather than time or geographical factors.

### 4.3 Is the evidence base robust against systematic publication bias?

The funnel plot and statistical tests were used to conduct a diagnostic assessment of the evidence base. The results showed that the reported outcomes were highly reliable. The funnel plot revealed the correlation between effect size and standard error. The distribution of 37 studies was approximately symmetrical around the pooled mean of 0.586, with most data points falling within the pseudo 95% confidence interval, forming a typical “inverted funnel” shape, which is a characteristic of a robust meta-analysis sample. Although there were some outliers at the higher end of the effect size, there was no significant asymmetry overall - the Egger linear regression results confirmed this judgment. Asymmetry is a typical manifestation of publication bias, so the absence of significant asymmetry means that the risk of publication bias is very low. This stability further indicates that the benefits observed from AI-assisted teaching are not just the selective reporting of positive results, but reflect a genuine improvement in teaching effectiveness. Therefore, the research results on intelligent teaching assessment, personalization, and adaptive learning can be regarded as a reliable reflection of the current state of the field, providing a reliable basis for policy-making.

### 4.4 Theoretical elaboration

By integrating educational psychology, technological innovation theory, and instructional design theory, a comprehensive framework can be established, which can further extend the theoretical explanation of AI teaching in teacher teaching reform. Bandura’s self-efficacy theory and social cognitive theory indicate that AI can reduce teachers’ repetitive tasks, such as homework grading and routine assessment, and improve teachers’ cognitive efficiency, teaching behavior, and their emotional confidence in the reform, which aligns with the core viewpoint of this theory, that is, technical assistance can enhance individual efficacy. The smaller pretest-posttest effect size reflects stricter baseline control, which conforms to the logic of accurate effect estimation in experimental psychology. The larger posttest-only effect size demonstrates the impact of immediate intervention. The pretest-posttest design can more accurately estimate the actual effect, which is consistent with the dose-effect theory of psychological intervention and the theory of intervention effectiveness. The situational specificity theory indicates that the effect of AI has high heterogeneity, that is, the I^2^ value is high, which depends on the type of AI, teachers’ digital capabilities, and teaching context. Different types of AI, such as generative, decision-assistance, and intelligent assessment, respectively support personalized teaching, data-driven teaching, and the optimization of teaching assessment. This is in line with the technology acceptance model and the technology usability theory, because the type of technology determines its impact on psychology and behavior. The result expectancy theory and reinforcement and feedback theory show that the direction of research results, that is, positive, negative, or mixed, is related to the effect size, reflecting the researcher’s bias, which is consistent with psychological reports and confirmation bias. The classical test theory ensures the stability of the effect because the confidence interval boundaries are related to the effect size. This correlation reflects the joint effect of measurement accuracy and research quality on intervention stability, linking measurement accuracy, research quality, and intervention stability. The sample statistics theory supports the robustness of large samples, and the larger sample size can better reflect the actual overall effectiveness of AI. Robustness indicates that the research results are closer to the overall real situation, thereby enabling the formulation of more targeted application strategies and implementing differentiated applications based on the type of AI and teaching context, thereby promoting reform. Evidence-based practice (EBP) confirms that AI has stable and positive effects, laying an evidence foundation for teaching reform. The precise intervention theory proposes to conduct differentiated design based on the type of AI to achieve precise assistance. The comparability theory tends to use the pretest-posttest design to conduct more rigorous causal inference. AI prompts a reconfiguration of the teacher’s role, from a knowledge disseminator to a learning guide, and promotes the redesign of the teaching process, forming an intelligent teaching model of human-machine collaboration. Teachers’ digital capabilities are the key to the effectiveness of AI, supporting the digital transformation of teaching. This comprehensive theory expands and enriches educational technology theory and can provide guidance for the practical application of AI in teaching reform.

### 4.5 Policy implications and the future of human-AI collaborative teaching?

Based on the results of various studies, it can be concluded that there are some key policy requirements for promoting the digital transformation of teaching. Firstly, since the “type of AI” has a significant impact on teaching effectiveness, educational authorities should not only focus on general hardware procurement but also make strategic deployments and select specific AI models that are consistent with the course objectives. Secondly, significant differences were found in the research, indicating that teachers’ digital capabilities are crucial for the success of the reform. Policies need to prioritize the development of professional development programs, which should not be limited to basic technical skills but should enable teachers to master human-machine collaborative teaching, that is, teachers should have the ability to interpret data dashboards and be able to make high-level teaching judgments based on algorithm recommendations. Thirdly, the reconfiguration of the teacher role needs to be formally recognized within the institutional framework (Shabani et al., 2010). Teachers should be given the role of “learning architect,” which is responsible for designing learning paths and activities, using data-driven methods to promote personalized and adaptive learning, rather than being merely regarded as operators of automated systems. Finally, methodological variables have marginal significance, indicating that the rigor of methodology and sampling strategies will have an impact on the observed results. Therefore, in future research, the standardization of reporting and the rigor of experimental design should be strengthened to improve the comparability and reliability of the conclusions. By establishing an ecosystem that values technological innovation and also focuses on redesigning the teaching process in a humanistic way, all relevant parties can ensure that the potential of intelligent teaching models is fully realized, and thereby achieve a more equitable and efficient educational future.
